# Synergistic interactions of cadmium-free quantum dots embedded in a photosensitised polymer surface: efficient killing of multidrug-resistant strains at low ambient light levels

**DOI:** 10.1039/c9nr10421f

**Published:** 2020-04-20

**Authors:** Ethel G. A. Owusu, Elnaz Yaghini, Imad Naasani, Ivan P. Parkin, Elaine Allan, Alexander J. MacRobert

**Affiliations:** a UCL Division of Surgery and Interventional Science , University College London , Charles Bell House , 43-45 Foley Street , London W1 W 7TS , UK . Email: a.macrobert@ucl.ac.uk; b Materials Chemistry Research Centre , Department of Chemistry , University College London , 20 Gordon Street , London WC1H 0AJ , UK; c Department of Microbial Diseases , UCL Eastman Dental Institute , University College London , 256 Gray's Inn Road , London WC1X 8LD , UK; d Nanoco Technologies Ltd , 46 Grafton Street , Manchester M13 9NT , UK

## Abstract

Cadmium-free quantum dots (QD) were combined with crystal violet photosensitising dye and incorporated into medical grade polyurethane *via* a non-covalent dipping process known as ‘swell-encapsulation-shrink’. The antibacterial efficacy of the prepared quantum dot-crystal violet polyurethane substrates (QD + CV PU) was investigated under low power visible light illumination at similar intensities (500 lux) to those present in clinical settings. The antibacterial performance of QD + CV PU was superior to the constituent polymer substrates, eliminating ∼99.9% of an environmental *P. aeruginosa* strain, a clinical *P. aeruginosa* strain from a cystic fibrosis patient and a clinical *E. coli* strain. The nature of the reactive oxygen species (ROS) involved in antibacterial activity of the QD + CV PU surface was investigated using ROS inhibitors and time-resolved optical spectroscopy. The photo-physical interactions of the green-emitting QDs with CV lead to a combination of Type I and II electron transfer and energy transfer processes, with the highly potent ROS singlet oxygen playing a dominant role. This study is the first to demonstrate highly efficient synergistic killing of clinical and environmental strains of intrinsically resistant and multi-drug resistant Gram-negative bacteria using light-activated surfaces containing biocompatible cadmium-free QDs and crystal violet dye at ambient light levels.

## Abstract 2

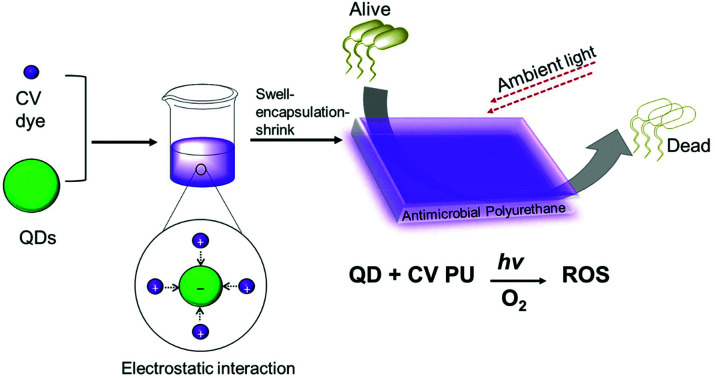
Polyurethane incorporated with cadmium-free quantum dots and crystal violet dye kill >99.9% of multi-drug resistant and intrinsically resistant clinical strains of bacteria under ambient light irradiation.

## Introduction

The development of antimicrobial resistance (AMR) is a natural process that has occurred long before the introduction of antibiotics to healthcare.^1^ However the overreliance and misuse of antibiotics has significantly accelerated the development and spread of bacteria resistance. AMR is now one of the biggest crises facing public health globally, estimated to cost $100 trillion and cause 10 million deaths annually by 2050.^2^ Resistance to antibiotics not only cripples the ability to fight common infections but also undermines the ability to perform several life-saving procedures which carry a high infection risk such as chemotherapy and organ transplantation.^3^ AMR is particularly problematic in some Gram-negative bacterial pathogens which are resistant to commonly available antibiotics and for which therapeutic options are severely limited, for example healthcare-associated *Pseudomonas aeruginosa* (*P. aeruginosa*) and Enterobacteriaceae.^4^ At the EU/EEA level, the European Centre for Disease Control reported that 30.8% of *P. aeruginosa* infections were resistant to at least one antibiotic treatment and more than half (58.2%) of *Escherichia coli* (*E. coli*) infections were resistant to at least one of the antibiotics usually administered for treatment.^5^

The increase in antimicrobial resistance in bacteria has led to increased efforts to identify new antimicrobial therapies. Therapies that effectively treat multi-drug resistant (MDR) bacterial infections as well as strategies that prevent and control infections are both important in reducing the impact of AMR. In the hospital, it has been shown that surfaces play a major role in the transmission of infections^6^ and moist environments such as hospital sinks, drains and faucets serve as environmental reservoirs of *Pseudomonas* spp. and Enterobacteriaceae, with many hospital outbreaks being traced back to these surfaces.^7^ Several studies have demonstrated the successful incorporation of photosensitisers (PS) and other light-activated antimicrobials into polymers for use in hospital surfaces to reduce nosocomial pathogen levels.^8^–^12^ Antimicrobial photodynamic therapy (aPDT) and other light-activated antimicrobial therapies, by definition, require a light source to provide the energy necessary to initiate the formation of reactive oxygen species (ROS) which are toxic to bacteria but have significantly lower toxicity to mammalian cells. While the energy required for photosensitisers differs according to molecular structure, in all cases, it is ideal to have a light source with strong light output however high intensity light sources may pose health risks.^13^,^14^ Further, the acquisition, implementation and maintenance of specialised lighting sources for aPDT such as lasers and wavelength-filtered light sources institution-wide may be impractical due to high costs or low accessibility and availability.^15^ Thus, an important yet often overlooked criterion for light-activated antimicrobial surfaces is the need to be effective under light intensities used in the environments for which they are designed. In the hospital setting, general lighting criteria (for example, in wards, reception areas and treatment rooms) to which hospitals must conform often require light intensities less than 1000 lux.^16^ At these low light levels, PDT is often ineffective, resulting in low antimicrobial activity.

Additionally, aPDT mediated by conventional dyes suffers from inherent limitations, particularly narrow absorption which limits suitable light sources for excitation. Quantum dots (QDs), semiconductors with sizes on the nanoscale, are attractive as photosensitisers for aPDT due to unique size-dependent spectral properties, bright luminescence with narrow symmetric bands, strong, broad-band absorption and high photo-stability. Combining QDs and PS is an attractive approach in aPDT as QD-PS complexes can overcome critical limitations of conventional PS and exploit the advantages of QDs, resulting in increased ROS production, specifically of singlet oxygen, *via* Förster Resonance Energy Transfer (FRET).^17^–^21^ However, the majority of QDs available are cadmium-based, restricting their translation to clinical use due to concerns over possible release of toxic ions.^22^,^23^ Cadmium-free QDs have been developed, including indium-based QDs, to alleviate these concerns and render the QDs biocompatible.^24^–^26^

Herein, we present novel light-activated polyurethane surfaces embedded with indium-based cadmium-free quantum dots and crystal violet dye and evaluate their antibacterial effectiveness against environmental and clinical isolates of Gram-negative *E. coli* and *P. aeruginiosa* pathogens. *E. coli* and *P. aeruginosa* colonise hospital water environments and there is opportunity for genetic exchange between them (with the potential for exchange of antibiotic resistance genes and virulence factors).^27^ In addition, biofilms formed by these bacteria are resilient, difficult to eradicate and are a common source of many persistent and chronic infections.^28^,^29^ Therefore, use of a material able to reduce colonisation by these bacteria would reduce biofouling, limit the opportunity for antibiotic resistance gene transfer and also decrease the transmission of hard-to-treat Gram-negative hospital-acquired infections.

Furthermore, the nature of the ROS involved in antibacterial activity of the quantum dot-crystal violet poluyrethane (QD + CV PU) surface is investigated using microbiological scavenger assays and optical spectroscopic techniques. Previously, these materials have been shown to be effective against multi drug-resistant MRSA and *E. coli* following application of strong light intensities (>6000 lux).^10^ This study in contrast demonstrates highly efficient photo-killing of clinical and environmental strains of Gram-negative bacteria by cadmium-free QD-containing antimicrobial surfaces activated at 500 lux, which is comparable to light intensities from ambient lighting found in general hospital environments.

## Results & discussion

### Characterisation

**Fig. 1:**
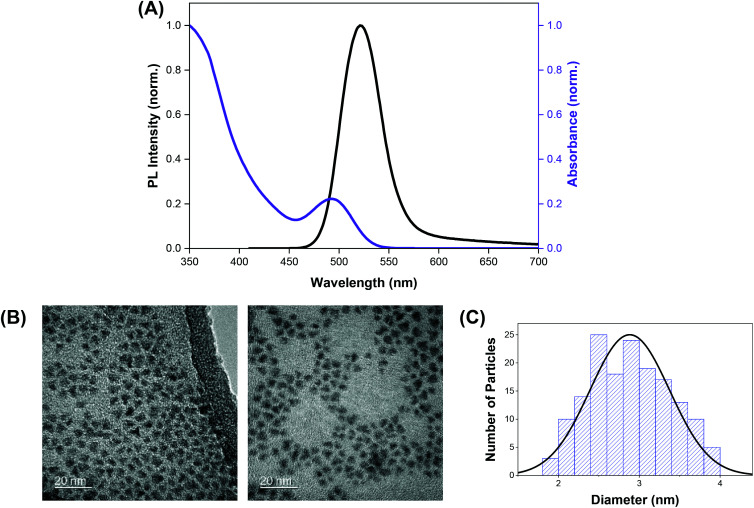
(A) Normalised absorption (purple trace) and emission (black trace) spectra of QDs. (B) High resolution TEM image of the green-emitting QD nanoparticles. (C) Size distribution of QD nanoparticles from TEM.

The indium-based QDs (CFQD® nanoparticles) were synthesised *via* a proprietary molecular seeding procedure,^30^ and showed broad absorption spectral features with the first excitonic peak centred at 493 nm. The emission spectrum of the QDs was found to be narrow with an emission peak at 520 nm and a full width at half maximum (fwhm) of 46 nm (Fig. 1A). HR-TEM images indicate that the green-emitting QDs were small, highly crystalline and monodisperse with a narrow size distribution and average particle size of 2.9 ± 0.5 nm (Fig. 1B and C).

Crystal violet (which is also known as gentian violet) is a highly coloured triphenylmethane dye with an extensive history as an anti-bacterial and anti-fungal agent. Due to the bacteriostatic activity of crystal violet, particularly against Gram-positive microorganisms, CV was employed in the treatment of wide range of diseases including oral candidiasis (thrush), eczema and as a blood additive to prevent transmission of Chagas’ disease from the early 1900s right through to the first half of the 20^th^ century.^31^–^34^ However, crystal violet fell out of favour with the clinical community in the 1940s following the discovery of antibiotics. With the emergence of antibiotic resistance, there has been a resurgence in interest in the use of crystal violet and other photosensitisers for clinical use. CV is inexpensive, readily available and chemically stable.^33^ Trials using CV have shown no or very mild adverse effects and no cases of cancer have been definitively linked to CV despite more than a century of use.^32^,^35^,^36^ FDA-approval was granted in 2003 for the use of CV in a bacteriostatic foam formulation for treatment of a range of wound types, and more recently for antibacterial dressings including incorporation in a polyurethane foam agent.^37^

**Scheme 1:**
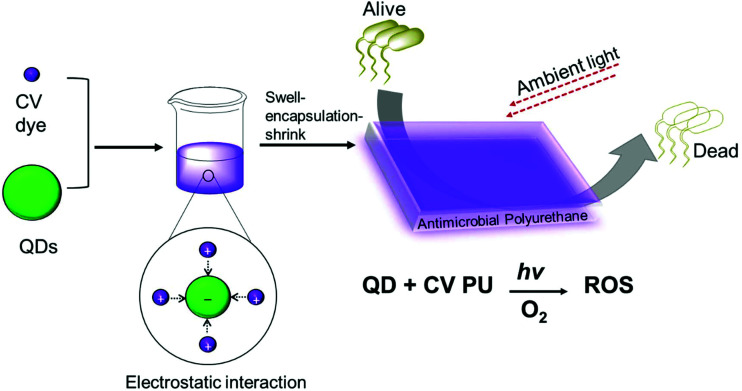
Preparation of antibacterial polyurethane substrates containing cadmium-free quantum dots and crystal violet.

Quantum dot and crystal violet encapsulated medical grade polyurethane polymers were developed using a one-step dipping strategy as described previously (Scheme 1).^10^ To facilitate simultaneous incorporation and co-localisation of the QDs and CV, the green-emitting QD nanoparticles and photosensitiser were combined in a miscible 1 : 1 cyclohexane/dichloromethane (Cy/DCM) solvent system. As the QDs were soluble in Cy whereas CV was not directly soluble, CV was initially dissolved in DCM prior to mixing with the QDs suspended in Cy. Polymer materials were immersed in the mixture for 24 h to facilitate optimal incorporation to achieve high ROS generation. Subsequently, the polymer substrates were air-dried and washed a minimum of three times to reduce CV leaching from the polymer over long periods of exposure to infectious microorganisms. QD photoluminescence and CV fluorescence measurements after modification of the polymer materials verified uptake of the QDs and/or CV materials.

### Antimicrobial testing

Antimicrobial tests were conducted on the polyurethane substrates incorporated with a combination of QDs and CV (QD + CV PU) against recent environmental and clinical isolates of *P. aeruginosa* and *E. coli*: *P. aeruginosa* P12, P1068 and *E. coli* 1030 under 500 lux white light irradiation for up to 24 h irradiation. As controls, solvent-treated polyurethane (control PU), QD-incorporated polyurethane (QD PU) and CV-incorporated polyurethane (CV PU) were also tested against the micro-organisms. Light intensity was carefully selected and limited to a maximum of 500 lux as this was close or equivalent to required lighting conditions for a wide range of settings and treatments in the hospital including general nursing care at bedsides (300 lux), clinical beds (400 lux), reception desks (500 lux), laboratories (500 lux), scrub-up areas (500 lux), anaesthesia rooms (500 lux) and examination or treatment at bed positions (1000 lux).^16^

*P. aeruginosa* is a non-fermentative Gram-negative bacterium common in aquatic environs in nature. It is an opportunistic pathogen for human, animals and plants, and is a major cause of infection in hospitalised patients. It is a common coloniser of moist environments within the hospital including sinks, sluices and showers and these can act as sources of infection during outbreaks.^38^,^39^ It commonly causes hospital-acquired pneumonia (including ventilator-associated pneumonia), bloodstream and urinary tract infections. Management of *P. aeruginosa* in hospitals and institutional environments is difficult because of its versatility and inherent tolerance to many detergents, disinfectants and antimicrobial compounds. Furthermore, treatment is being rendered increasingly challenging due to the emergence and spread of resistance to the few therapeutic options available.^5^,^40^

**Fig. 2:**
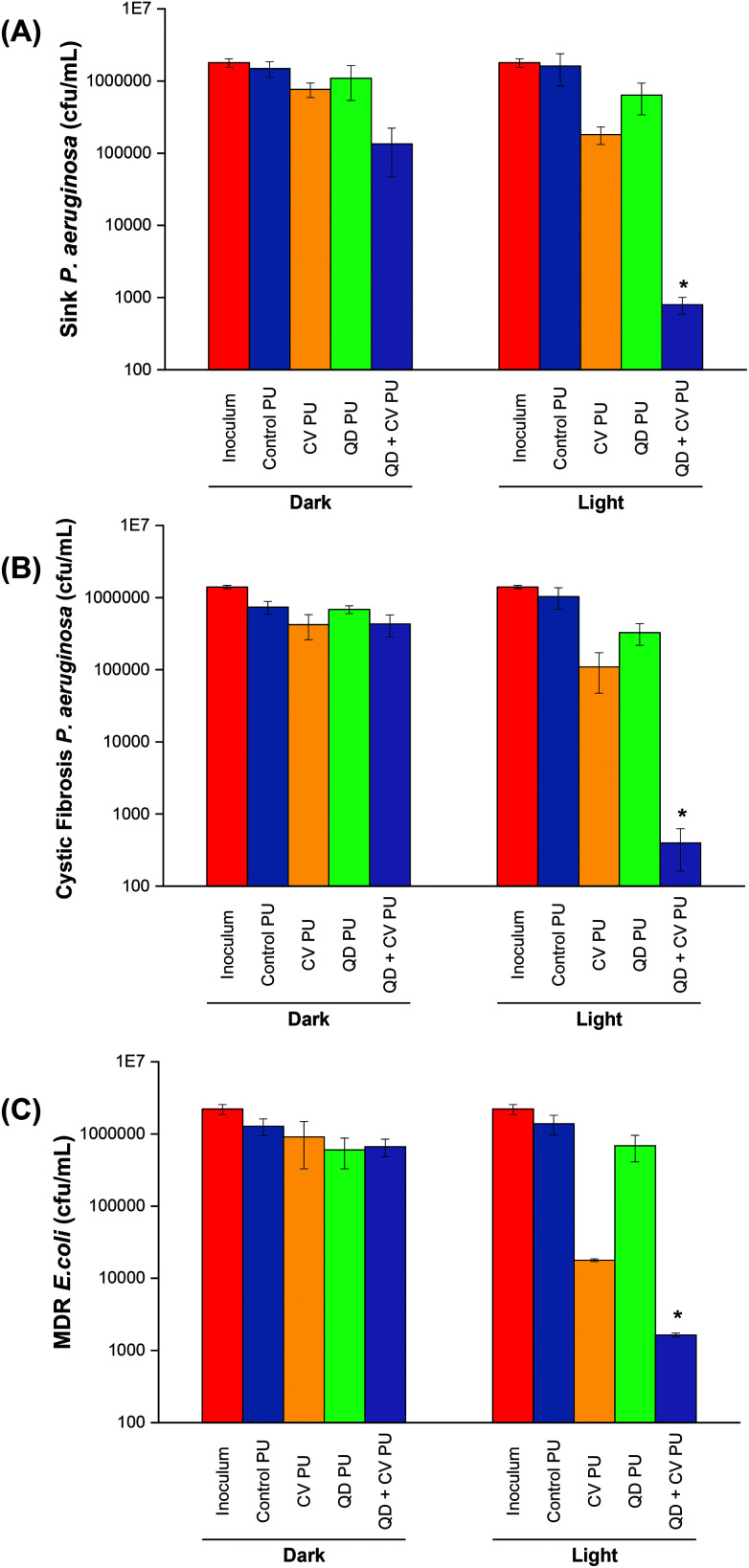
Viable counts of (A) environmental *P. aeruginosa* P12 on modified polyurethane surfaces after 24 h irradiation at 500 lux, (B) clinical *P. aeruginosa* P1068 on modified polyurethane surfaces after 24 h irradiation at 500 lux (C) multi-drug resistant *E. coli* 1030 on modified polyurethane surfaces after 18 h irradiation at 500 lux. * indicates that QD + CV PU has *P* ≤ 0.05 compared to CV PU.

*P. aeruginosa* P12, a generally sensitive isolate was recovered from a patient sink within an Intensive Care Unit at King's College Hospital, London. Environmental isolates, which are a known source of infection, are generally quite sensitive to antibiotics compared to patient isolates, suggesting that resistance develops after infection.^41^ The bactericidal efficiency of the various doped polyurethane against *P. aeruginosa* was investigated following either 24 h in the dark or 24 h exposure to a low intensity white light source with intensity averaging 499 ± 19 lux (Fig. 2A). Following 24 h dark incubation, there was no significant change in *P. aeruginosa* numbers on the control PU and QD PU substrates indicating no antibacterial activity, whereas CV PU and QD + CV PU samples showed a 0.3 log_10_ and 1.0 log_10_ reduction in bacterial numbers, respectively. Exposure to low intensity white light for a period of 24 h also showed no bactericidal activity on the control PU or QD PU but the addition of CV (CV PU) produced a 0.9 log_10_ reduction in bacterial numbers and when both quantum dot and CV were present in the polymer (QD + CV PU), the greatest bactericidal activity was observed with numbers of *P. aeruginosa* reduced by 3.3 log_10_ (99.95%; *P* = 0.03 compared to CV PU), following exposure to white light for 24 h.

Most cystic fibrosis (CF) patients suffer from chronic *P. aeruginosa* infections that are hard to treat due to resistance to current antibiotics, leading to respiratory failure and lung disease. These infections commonly occur in patient tissues or on the skin as well as the surfaces of medical devices such as joint replacements, indwelling venous and urinary catheters and ventilators and are transmitted by close contact to contaminated surfaces. Hygienic measures to prevent colonisation of CF patients include high-level disinfection of non-disposable equipment and surfaces.^42^–^44^ The light-activated combinatorial therapy in this study provides an effective means to reduce levels of *P. aeruginosa* on surfaces.

The antimicrobial activity of the substrates against *P. aeruginosa* P1068, a clinical isolate from a CF patient, was also tested (Fig. 2B). No statistically significant difference was observed for any of the substrates in the dark. Upon 500 lux light illumination, no bactericidal effect was elicited by either control PU and QD PU. However, under low level light illumination, CV PU induced a 0.9 log_10_ reduction in cystic fibrosis *P. aeruginosa* numbers and the combination of QD + CV in the surface resulted in a 3.4 log_10_ kill of this patient isolate of *P. aeruginosa*, corresponding to a 99.96% efficiency (*P* = 0.025). The significantly greater efficacy of the combination of CV and QDs in PU revealed that a synergistic interaction potentiated the antimicrobial effect. These results differed to some degree with our previous study using red-emitting QDs where much higher light intensities were employed to achieve bacterial kill.^10^ Under those conditions the CV dye contributed significantly to the observed kill, whereas in the present study the contribution of CV to the overall kill was relatively low.

*Escherichia coli* is a Gram-negative bacterium that normally lives in human and animal intestines as it is needed for bowel function and is an important part of a healthy human intestinal tract. However, it is also a common cause of hospital-acquired urinary tract infections, septicaemia and meningitis. Like *P. aeruginosa*, *E. coli* and other members of the Enterobacteriaceae thrive in moist hospital environments, which can act as sources of infection.^45^,^46^ AMR in *E. coli* develops either through mutations or by acquisition of mobile genetic elements encoding resistance mechanisms, such as the production of beta-lactamases and extended spectrum beta-lactamases (ESBLs), conferring resistance to beta-lactam antibiotics such as ampicillin, amoxicillin and third generation cephalosporins. Carbapenems are a class of antibiotics that can inhibit the effect of ESBLs and therefore are reserved as last resort antibiotics for severe MDR infections. However, the horizontal acquisition of carbapenem resistance mediated by a range of carbapenemase enzymes is a growing problem and, may confer resistance to all available beta-lactam antibiotics.^5^

The activity of the substrates against *E. coli* 1030, a carbapenemase-producing clinical strain was also analysed (Fig. 2C). Following 18 h incubation in the dark, there was no significant antibacterial effect on *E. coli* from any of the PU substrates. On the other hand, 18 h low level light irradiation resulted in a 1.8 log_10_ reduction in the numbers of *E. coli* for the CV PU substrates and a 2.9 log_10_ reduction in bacterial numbers for the QD + CV PU substrates (99.88%, *P* = 0.001 compared to CV PU). Again, the control PU and QD PU substrates showed no significant bactericidal activity.

We tested recent environmental and clinical isolates of *E. coli* and *P. aeruginosa* as these are major problems in healthcare currently. The decision to use recent isolates from clinical environments was taken because it is well known that strains acquired from culture collections may no longer be representative of wild strains. To compare the activity of the new material with previously published materials, we have carried out recent studies with polyurethane containing crystal violet and 3–4 nm zinc oxide (ZnO) nanoparticles. Illumination at 500 lux of CVZnO substrates showed comparable antimicrobial effects against *E. coli* and *P. aeruginosa*, although the *P. aeruginosa* strain employed in the study was a laboratory isolate.^47^ Another approach using polymer surfaces with antimicrobial activity was undertaken by Boyer *et al.* An antimicrobial polymer consisting of oligoethylene glycol, hydrophobic ethylhexyl, and cationic primary amine groups was effective against *P. aeruginosa*. However, good bacteriostatic activity was achieved by combining the antimicrobial polymer with the antibiotic doxycycline. We note that the strains used were laboratory strain PA01 and isolates from keratitis, which are likely to exhibit very different responses from the CF and environmental isolates that we used herein.^48^

Since *E. coli* and *P. aeruginosa* were both Gram-negative, photo-killing efficacies were expected to be comparable as they shared similar structural characteristics. However, for the same percentage antibacterial activity, a longer irradiation times were required for *P. aeruginosa* strains. This may be attributed to the fact that *E. coli* possesses both non-specific large general porins and substrate-specific channels on its outer membrane, whereas *P. aeruginosa* does not display such large general porins, allowing only specific diffusion of various small molecules.^49^ The lack of general porins in *P. aeruginosa* meant that penetration of ROS was reduced compared to *E. coli*, resulting in slower disinfection rates of *P. aeruginosa* by the QD + CV PU surface. By the same token, any leakage of CV and/or QD particles from the QD + CV PU surface would have had a lower lethal effect on *P. aeruginosa*. Furthermore, *P. aeruginosa* may be capable of withstanding highly oxidative environments for longer periods of time due to its lower cell permeability (∼100-fold lower cell permeability than *E. coli*).^50^ Moreover, *P. aeruginosa* adaptive mechanisms such as the ability to produce pigments such as pyomelanin have been shown to confer a degree of tolerance to oxidative stress generated by photo-activation of photosensitisers.^51^,^52^

Against each bacterial strain, polyurethane containing only QDs (QD PU) showed no significant bactericidal activity. This was previously noted in our other studies and was attributed primarily to the limitations in nanoparticle uptake by the polymer (*via* the non-covalent incorporation method employed) and also, QD absorption being strongest at UV wavelengths and lower at the wavelengths emitted by the light source used.^53^ Additionally, a small but significant reduction of *P. aeruginosa* on CV PU in the absence of illumination was observed. This limited dark toxicity against the bacterium was likely a result of CV's intrinsic antimicrobial properties that, for decades prior to the discovery of antibiotics, made CV an effective broad-spectrum therapeutic agent.^33^,^54^ Irradiation of QD + CV PU resulted in markedly increased bactericidal activity compared to irradiated CV PU. This implied that the interaction between QDs and CV presented additional sources of ROS production *via* Förster resonance energy transfer (FRET) and/or photoelectron transfer (PET).

### Spectroscopic analysis of QD + CV complex

**Fig. 3:**
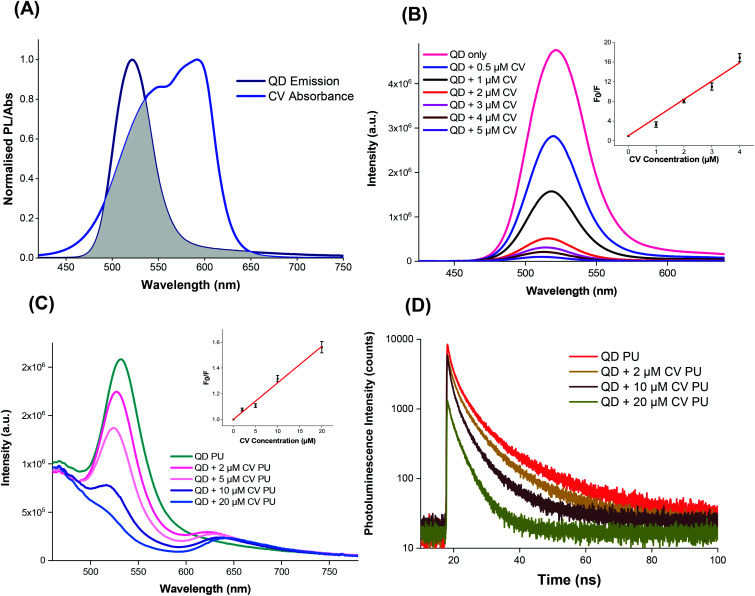
(A) Spectral overlap of QD emission and crystal violet absorbance. PL excitation at 400 nm. (B) Emission of QD with increasing CV concentration in solution at 400 nm excitation. Inset: S–V relationship of QD quenching *vs.* CV concentration in solution at 500 nm. (C) Emission of QD alone or in combination with increasing concentrations of CV into polyurethane (excitation at 400 nm). Inset: Stern–Volmer plot of QD fluorescence quenching ratio *vs.* CV concentration in polyurethane at 500 nm. (D) Time-resolved emission of QD alone or in combination with increasing concentrations of CV in polyurethane.

The QDs were capped with ligands featuring electronegative groups, enabling formation of electrostatically bound complexes with CV which is cationic in the aprotic solvent system under study. Thus the complex formed may allow the occurrence of short-range energy transfer pathways.^55^,^56^ The ability of the QDs and CV to function as a FRET donor–acceptor complex was evidenced by the significant overlap of the QD emission with CV absorbance as illustrated in Fig. 3A.^57^,^58^ The degree of spectral overlap between acceptor absorption and the donor emission was calculated as done previously.^10^ The overlap integral *J*(*λ*) of the QD + CV complex was calculated to be 8.04 × 10^13^ nm^4^ M^–1^ cm^–1^, corresponding to good spectral overlap of the QD + CV complex, verifying the feasibility of the occurrence of non-radiative energy transfer from photo-excited QD donor to the ground state CV acceptor.^57^ From the overlap integral, the Förster radius (*R*_0_), the distance at which the FRET efficiency is 50%, was subsequently derived as 3.05 nm.^59^ So we propose in this case that the net effect was that the QDs enabled better light harvesting from a white light source and facilitated radiationless transfer of this energy to the dye thereby inducing greater ROS production and enhanced bacterial kill through a synergistic mechanism. This scheme was evaluated using steady-state and time-resolved luminescence measurements, as shown in Fig. 3.

The photoluminescence of the QDs was efficiently quenched by the addition of CV in Cy/DCM solution (Fig. 3B) however no concomitant increase in CV emission was observed in solution as would be expected in FRET. This may be due to the low fluorescence yield of the CV dye in a low viscosity microenvironment caused by formation of twisted intramolecular charge transfer (TICT) states which rapidly relax to the ground state.^60^ However when incorporated in a rigid polymer, where viscosity was extremely high, intramolecular twisting to form TICT states was restricted and CV fluorescence was observed, as shown in Fig. 3C. In this microenvironment, the peak emission of the QDs decreased with the addition of increasing concentration of CV in the polymer. A concentration-dependent bathochromic shift in the CV emission peak was also observed which was ascribed to absorption of the shorter wavelengths of the CV emission causing suppression of the signal approaching to 600 nm. This phenomenon was also observed in polymer in the absence of QDs.^10^

Stern–Volmer plots of the fluorescence quenching *vs.* CV concentration in solution and polymer phases at 500 nm (where CV absorption is weaker) showed linear behaviour when *F*_0_/*F versus* [Q] is plotted, in the form:

1 *F*_0_/*F* = 1 + *K*_SV_ [Q]

where *F*_0_ and *F* are the fluorescence intensities (with the control background subtracted) in the absence and presence of quencher, *K*_SV_ is the Stern–Volmer quenching constant and [Q] is the quencher concentration (insets of Fig. 3B and C). The value of *K*_SV_ is derived as 3.6 × 10^6^ M^–1^.

For the solution phase studies, the quenching constant (*k*_q_) was derived by dividing the Stern–Volmer quenching constant by the PL lifetime of the excited QDs without addition of CV. Using bi-exponential fitting, the mean lifetime was 49 ns which gave *k*_q_ = 7.4 × 10^14^ M^–1^ s^–1^ for the QDs in solution; for polymer a nominal value of *k*_q_ was derived using the CV concentration present in the embedding solution and the mean PL lifetime of 12 ns of CV in the polymer, which gave *k*_q_ = 2.5 × 10^12^ M^–1^ s^–1^. The solution phase value of *k*_q_ on addition of CV, was several orders of magnitude higher than the diffusion controlled limit for bimolecular reactions (c. 10^10^ M^–1^ s^–1^), too high for collisional quenching. Accordingly, CV must be bound to the QD surface and the resulting interactions within the QD + CV complex were predominantly static in nature.^61^ We note that the value of *K*_SV_ was significantly higher than the value we measured for red-emitting indium-based QDs (4.4 × 10^5^ M^–1^) under the same experimental conditions showing that the photophysical interaction between green QDs and CV was stronger than the red QDs used previously.^10^ A possible explanation is that this effect results from the smaller mean diameter of the green QDs (2.9 nm *versus* 3.6 nm for the red QDs), in accord with the quantum confinement effect on the band gap. The larger surface to volume ratio of the green QDs will confer a higher ratio of the electronegative capping ligands per unit inorganic mass, thereby enhancing the propensity for electrostatic surface binding of the cationic crystal violet molecule. Since the solution employed for incorporation of the QDs and CV into the polymer contained a mixture of the two moieties, strong binding should promote direct uptake of QD + CV complexes into the polymer. Under this scenario, quenching of the QD PL should therefore occur in the polymer when co-administered with CV.

The photoluminescence lifetime of the QDs decreased with increasing CV concentration (Fig. 3D), which was consistent with the QD emission quenching observed (Fig. 3C), and confirmed that the QD must be in close proximity to CV within the polymer. Although no concomitant generation of CV fluorescence was apparent in solution, the presence of CV emission was evident in the polymer. Generally, shortened PL lifetimes may result from energy transfer, electron transfer and non-luminescent exciplex formation although the lack of change in the shape of the absorption spectra appeared to rule out exciplex formation. The large *J*(*λ*) integral along with the improvement in antimicrobial activity upon combining of QDs and CV favoured the occurrence of FRET and singlet oxygen formation however the relative contributions of FRET *vs.* electron transfer was unclear.

In addition to FRET, photoelectron transfer (PET) may also occur leading to generation of reactive oxygen species, principally superoxide radical anions. Photo-excited indium-based QDs have been shown to produce superoxide^62^ but evidently in our studies using low ambient light levels, the yield of ROS from photo-excited QDs was insufficient to elicit a significant antimicrobial effect. PET was also possible between the QD and CV when in close proximity. For example, photoexcitation of either the QDs or CV could result in electron donation to CV, which is a good electron acceptor, and the reduced CV radical may subsequently interact with oxygen to form superoxide, as has been previously demonstrated by various groups.^63^–^65^

### Studies of mechanistic mode of action: role of reactive oxygen species

The exact mode of action of the QD + CV polymer substrates was not yet fully understood however, since the addition of QDs enhanced the light-activated bactericidal effects of CV alone, this suggested that the mechanism of QD + CV PU operated *via* a Type I and/or Type II pathway. Type I mechanisms involve photo-electron transfer (PET) to generate free radicals such as superoxide anions and hydroxyl radicals. The Type II pathways involve direct energy transfer from the photosensitiser triplet state to molecular oxygen generating highly reactive singlet oxygen (^1^O_2_). Singlet oxygen plays a dominant role in photo-activity of surfaces, in agreement with studies demonstrating the effectiveness of ^1^O_2_ in killing bacteria and evidence of application of FRET for improved antimicrobial activity of light-activated surfaces. Extensive work has been performed on determining the lifetime of ^1^O_2_ in media. Radiative decay of ^1^O_2_ to the triplet ground state of oxygen ^3^O_2_ is a strongly forbidden process hence singlet oxygen has a long phosphorescence lifetime in the gas phase. However, in condensed media, this lifetime is significantly shortened by physical quenching to the millisecond and microsecond range, depending on the solvent.^66^

**Fig. 4:**
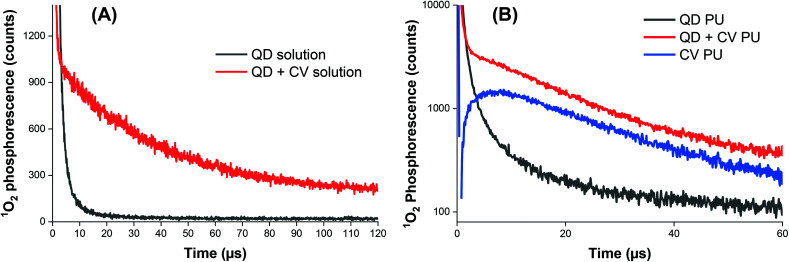
Time-resolved singlet oxygen phosphorescence recorded at 1270 nm following pulsed laser irradiation of (A) QD solution (black line) and QD + CV combination (red line). (B) Modified polyurethane containing QD only (black line), CV only (blue line), and a combination of QD and CV (red line). The traces have been subtracted from the control (untreated polyurethane).

Using ^1^O_2_ phosphorescence measurements, further analysis of ^1^O_2_ production by the QD + CV complex was carried out in both solution phase and upon incorporation of the QD + CV combination in polymer. The decay curve of the QD + CV complex in a solvent system of 1 : 1 cyclohexane/DCM (Cy/DCM) was fitted to a single exponential (eqn (2)):

2 *I*_t_ = *A*_1_*e*^(–*t*/*τ*_1_)^

where *I*_t_ is the phosphorescence intensity, and *A* and *τ* are the fractional amplitude and emission lifetime respectively (Fig. 4A). An average amplitude weighted ^1^O_2_ lifetime of 3.79 × 10^–5^ s was determined and subsequently, the rate constant of decay of ^1^O_2_ in 1 : 1 Cy/DCM solvent was determined to be 2.64 × 10^4^ s^–1^ from the reciprocal of this value. ^1^O_2_ photo-generated *via* FRET can be quenched by both Cy and DCM and in a mixed system of these 2 solvents, the decay rate constant of ^1^O_2_ (*k*_d_) may be expressed as follows:

3 *k*_d_ = *x*_1_*k*_1_ + *x*_2_*k*_2_

where *x*_1_ and *x*_2_ are the mole fractions of Cy and DCM in the systems respectively and *k*_1_ and *k*_2_ are the ^1^O_2_ decay rate constants from the reported ^1^O_2_ lifetimes in each solvent.^67^ In 1 : 1 Cy/DCM, *x*_1_ = *x*_2_ = 0.5. The literature reported ^1^O_2_ lifetimes as 2.3 × 10^–5^ s in Cy and 9.9 × 10^–5^ s in DCM, yielding values of 4.3 × 10^4^ s^–1^ and 1.0 × 10^4^ s^–1^ for the ^1^O_2_ decay constant in Cy and DCM respectively.^68^,^69^ Applying eqn (3), this corresponded to a literature ^1^O_2_ decay rate constant of 2.68 × 10^4^ s^–1^ of in 1 : 1 Cy/DCM. This value was in excellent agreement (within 2%) with the experimental ^1^O_2_ decay rate constant of 2.63 × 10^4^ s^–1^, indicating that in solution, ^1^O_2_ quenching occurred only through interaction with solvent vibrational modes and not the CV or QD solute.

Phosphorescence measurements of ^1^O_2_ were also carried out after incorporating QDs and/or CV into polyurethane (Fig. 4B). CV PU (blue line) showed an initial characteristic rise in signal, governed by the rate of conversion of triplet oxygen to singlet oxygen by the quenching of excited CV to its ground state. This feature was followed by an exponential decay, dictated by the subsequent quenching of singlet oxygen by the medium (polymer matrix) based on our previous studies.^70^ The very short-lived initial spike <1 μs corresponded to CV fluorescence and was therefore disregarded in subsequent analyses.

Fitting the initial rise to a bi-exponential function:

4 *I*_t_ = *A*_1_*e*^(–*t*/*τ*_1_)^ + *A*_2_*e*^(–*t*/*τ*_2_)^

with a negative *A*_2_ component – corresponding to the rise in signal, gave a rise-time (*τ*_2_) of 3.9 μs. The decay component was fitted to a mono-exponential decay (eqn (2)) and the lifetime of ^1^O_2_ in the CV PU was found to be 38 μs, in good agreement with the reported ^1^O_2_ lifetime of CV embedded in a similar polymer (silicone, 40 μs).^70^

When only QDs were present in the polymer matrix (black line), an initial signal of high intensity was observed. The signal was relatively short-lived, lasting about 10–15 μs and was caused by the long wavelength tail of QD photoluminescence, which albeit very weak in intensity was still relatively intense *versus* the very weak ^1^O_2_ phosphorescence intensity detected at 1270 nm. An exponential decay was not observed for QD PU as the generation of ^1^O_2_ upon photo illumination is an energetically unfavourable process in indium-based QDs.^62^

The combination of QDs and CV in the polymer matrix (red line) gave rise to a similar decay profile in signal consistent with singlet oxygen generation but markedly different dynamics at short times <5 μs. In contrast to CV alone, a short-lived spike in signal was observed which corresponds to residual QD emission. Thus, in the presence of CV, some quenching of QD emission occurred in PU which can be ascribed to a combination of FRET and/or photoelectron transfer to generate either singlet oxygen or superoxide. This was possible since there is still residual QD absorption at 532 nm (the laser excitation wavelength) and FRET-induced singlet oxygen generation could contribute to the observed signal. However, CV absorption at 532 nm is still relatively strong compared to its peak absorption near 600 nm therefore some of the signal originates from CV bound within the matrix that is remote from the QD and possibly from direct excitation of CV bound to the QD.

**Fig. 5:**
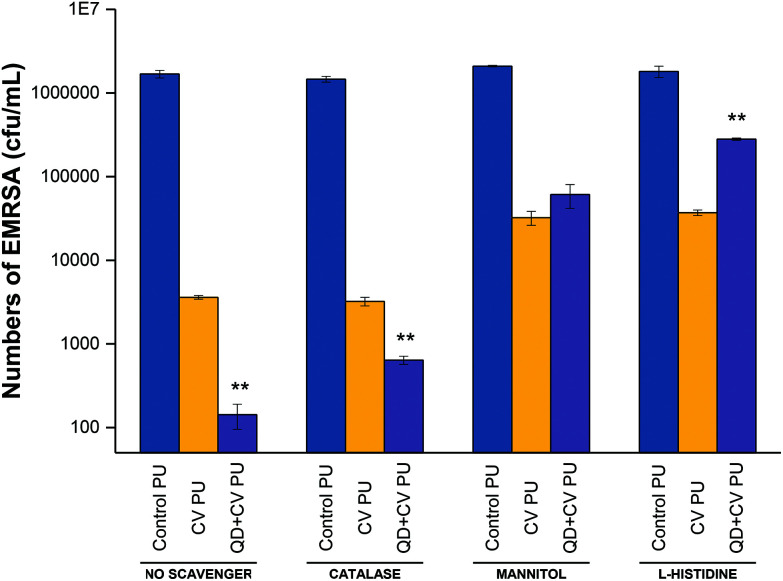
Photo-bactericidal activity of control PU, CV PU and QD + CV PU against EMRSA in the absence of ROS scavengers (‘no scavenger’) and in the presence of catalase (H_2_O_2_ scavenger), mannitol (hydroxyl scavenger) and l-histidine (^1^O_2_ scavenger). ** indicates that QD + CV PU has *P* ≤ 0.01 compared to CV PU.

To investigate the contribution of energy transfer to the system as well as how electron transfer was involved in photo-bactericidal activity, specific ROS scavengers were introduced into microbiological assays to identify the particular oxygen species produced by the surfaces. Scavengers of ROS formed from Type I and Type II mechanisms were added to the antimicrobial testing protocol to investigate their effect on the bactericidal performance (Fig. 5). A H_2_O_2_ scavenger (catalase), a hydroxyl radical scavenger (mannitol) and a ^1^O_2_ scavenger (l-histidine) were used in the microbiological investigation against EMRSA 4742. Catalase causes enzymatic conversion of H_2_O_2_ to H_2_O and O_2_, deactivating the ROS; mannitol effectively scavenges hydroxyl radicals (˙OH); and the aromatic amino acid, l-histidine, chemically reacts with ^1^O_2_ to form a number of oxidised products and effectively quench ^1^O_2_.^71^–^74^

Fig. 5 shows the bactericidal activity of control, CV, and QD + CV incorporated polyurethane samples against EMRSA 4742 upon white light irradiation, with the addition of catalase, mannitol and l-histidine. The experiment labelled “no scavenger” was not treated with any ROS quenchers, in order to demonstrate and compare the effect of adding each scavenger separately in each set of experiment.

Following irradiation, the bacterial numbers on the control polymer were not affected upon the addition of catalase, mannitol or l-histidine. On the other hand, bacterial numbers on CV PU samples were reduced to 2.6 log_10_ in the presence of catalase, 1.68 log_10_ in the presence of mannitol and 1.8 log_10_ in the presence of l-histidine, compared to a 2.8 log_10_ reduction when no scavenger was present. The addition of catalase to the QD + CV PU assay resulted in a 3.4 log_10_ reduction compared to the 4.2 log_10_ reduction in the experiment which did not include any quenchers. Furthermore, when mannitol was added, the bactericidal activity reduced to 1.5 log_10_ reduction and the addition of l-histidine reduced QD + CV PU activity to 0.8 log_10_. The significant difference in anti-bacterial action of QD + CV PU upon the addition of catalase and mannitol indicated that both H_2_O_2_ and ˙OH were formed by the substrate, respectively.

The presence of H_2_O_2_ likely resulted from the production of superoxide (O_2_˙^–^) by QD + CV PU light-activated system. Unlike superoxide, H_2_O_2_ is a non-radical derivative of O_2_ and thus less reactive and more stable with a longer half-life. In biological systems, superoxide is short-lived owing to its disproportionation to H_2_O_2_ either spontaneously or *via* endogenous superoxide dismutase (SOD), and scavenging by other substrates.^75^ The charge on the superoxide anion radical makes it unable to cross cellular membranes, except possibly through ion channels. In contrast, H_2_O_2_ has a longer biological lifespan than O_2_˙^–^, comparatively stable, and easily diffusible within and between cells.^76^ Since the main source of H_2_O_2_ was the disproportionation of superoxide (2O_2_˙^–^ + H^+^ → H_2_O_2_ + O_2_), it follows that the effect of catalase on the light-activated surface in this study directly correlated to the contribution of O_2_˙^–^ in the system. Since H_2_O_2_ and ˙OH are species formed from Type I reaction pathways, this proved that a Type I mechanism was involved in the light-activated lethal activity of QD + CV PU.

QDs strongly absorb energy at wavelengths shorter than their PL wavelength. As energy donors, they can undergo energy transfer with ground-state CV acceptors which subsequently interact with nearby oxygen molecules to form cytotoxic ^1^O_2_ species *via* the Type II process. Direct photoexcitation of CV is an alternative source of singlet oxygen also *via* the Type II process. Histidine is a well-known ^1^O_2_ scavenger that can directly react with ^1^O_2_ at a very high reaction rate.^77^ When histidine was introduced into the assay, it reacted rapidly with singlet oxygen thus reducing the oxidative stress encountered by the bacteria. The reduction in the antibacterial action of QD + CV PU in the presence of l-histidine was evidence for the operation of a Type II mechanism. While ^1^O_2_ may also be generated *via* direct excitation of CV, the addition of the ^1^O_2_ quencher affected CV PU to a lesser extent (activity dropped from 2.8 log_10_ with no scavenger to 1.8 log_10_ with l-histidine present) than QD + CV PU (activity dropped from 4.2 log_10_ to 0.8 log_10_ with l-histidine present). This indicated that: (1) QD + CV PU substrates produced more ^1^O_2_ than CV PU substrates and (2) ^1^O_2_ was generated through processes other than direct CV excitation. FRET was the other process by which ^1^O_2_ was formed.

Therefore, from the data, we conclude that the enhancement in bacterial activity when QDs were combined with CV was due to the occurrence of both Type I and II mechanisms. Type II mechanisms provided a greater contribution to the antibacterial effect as bactericidal action was most strongly attenuated in the presence of the ^1^O_2_ scavenger. In addition, comparing the effect of the ROS scavengers on CV PU to QD + CV PU, we observed that CV PU produced a minimal amount of H_2_O_2_ and significantly less ˙OH and ^1^O_2_ than QD + CV PU. As the ROS were responsible for lethal activity, this explained the lower activity of CV PU against *E. coli* and *P. aeruginosa*.

These data are in accord with other studies from our laboratory using nanoparticle/CV combinations. For example, Sehmi *et al.* studied ROS production when ZnO nanoparticles and CV photosensitising dye were incorporated into polymer (CVZnO) and irradiated at high light intensity levels (6600 lux) against laboratory strains of *E. coli* and *S. aureus*. Here, specific scavenger assays revealed that both Type I and II mechanistic pathways operated in the lethal activity of the CVZnO system, however Type II process provided a greater contribution.^12^ The present work shows that under white light of far lower intensities (13 times lower), Type I and II mechanisms still operate and result in efficient kill of intrinsically resistant and multi-drug resistant strains of Gram-negative bacteria.

## Conclusion

This study is the first to demonstrate highly efficient killing of a clinical strain of *P. aeruginosa*, an environmental strain of *P. aeruginosa* and a multi-drug resistant carbapenamese-producing *E. coli* strain using light-activated polyurethane surfaces containing cadmium-free QDs and CV photosensitiser. Significantly, the antibacterial activity was achieved under the same ambient white light intensities found in hospital treatment rooms and wards, which are much lower than direct sunlight. *P. aeruginosa* and *E. coli* biofilms are prevalent within the hospital environment, frequently colonise catheters and are associated with bacteraemia in hospitalised patients. Modifying the surfaces of biomedical devices by impregnation with cadmium-free QDs and CV is a potential strategy to prevent surface colonisation by planktonic bacteria and thus, prevent subsequent biofilm formation.

ROS scavenger studies revealed that Type I and Type II mechanisms are responsible for the antibacterial action of QD + CV PU surfaces and that the addition of QDs to CV enhances the photo-antimicrobial activity of CV in a synergistic interaction. Thus, the mechanistic pathways of the QD + CV PU materials are independent of any antibiotic resistance patterns, making them effective against both susceptible and drug-resistant bacterial pathogens. The combination of QDs that absorb light in the blue to green regions with a photosensitiser that absorbs in the red to the near-infrared regions ensures that these materials have good spectral coverage, enabling efficient light harvesting. This also lends flexibility to the materials since they can be activated with either a light source with a broad optical bandwidth (as is the case here) or a narrow band light source such as a laser/LED using fibre-optic light delivery, which can potentially be applied to *in situ* sterilization of indwelling devices such as endotracheal tubes and urinary catheters. In conclusion, QD + CV polymer materials made using biocompatible non-toxic QDs show great promise for use as self-disinfecting surfaces in the clinical environment.

## Methods and materials

### Synthesis of quantum dot nanoparticles

Quantum dot CFQD® nanoparticles based on indium (In) compositions were manufactured in the laboratories of Nanoco Technologies Ltd, Manchester, UK by means of proprietary synthetic procedures based on the patented molecular seeding process.^30^ Briefly, the preparation of crude indium-based quantum dots with emission in the range of 500–700 nm was carried out as follows: dibutyl ester (approximately 100 mL) and myristic acid (MA) (10.06 g) were added to a three-neck flask and degassed at ∼70 °C under vacuum for 1 h, after which, nitrogen was introduced and the temperature increased to ∼90 °C. Approximately 4.7 g of the ZnS molecular cluster [Et_3_NH_4_] [Zn_10_S_4_(SPh)_l6_] was added, and the mixture was stirred for approximately 45 min. The temperature was then increased to ∼100 °C, followed by the drop-wise additions of indium myristate (In(MA)_3_) (1 M, 15 mL) and tris(trimethylsilyl)phosphine ((TMS)_3_P) (1 M, 15 mL). The reaction mixture was stirred while the temperature was increased to ∼140 °C, at which point, further drop-wise additions of In(MA)_3_ dissolved in di-*n*-butyl sebacate ester (1 M, 35 mL) (left to stir for 5 min) and (TMS)_3_P dissolved in di-*n*-butyl sebacate ester (1 M, 35 mL) were made. The temperature was then slowly increased to 180 °C, and another round of dropwise additions of In(MA)_3_ (1 M, 55 mL) followed by (TMS)_3_P (1 M, 40 mL) was made. By addition of the precursor in this manner, indium-based particles with an emission maximum gradually increasing from 500 nm to 720 nm were formed. For the present study, the reaction was stopped at this stage when the desired emission maximum at ∼500 nm was obtained and left to stir at the reaction temperature for half an hour.

After this period, the mixture was left to anneal for up to approximately 4 days (at a temperature ∼20–40 °C below that of the reaction). A UV lamp was also used at this stage to aid in annealing. The particles had a final emission peak at 520 nm and were isolated by the addition of dried degassed methanol (∼200 mL) *via* cannula techniques. The precipitate was allowed to settle and then methanol was removed *via* cannula with the aid of a filter stick. Dried degassed chloroform (∼10 mL) was added to wash the solid then the solid was left to dry under vacuum for 1 day. This procedure resulted in the formation of indium-based nanoparticles on ZnS molecular clusters. Washing in dilute hydrofluoric acid (HF) increased the quantum efficiency of the resultant indium-based nanoparticles.

Growth of a ZnS shell: HF-etched indium-based core particles (20 mL portion) was dried in a three-neck flask. 1.3 g of myristic acid and 20 mL di-*n*-butyl sebacate ester were added and degassed for 30 min. The solution was heated to 200 °C, and 2 mL of 1 M bis(trimethylsilylmethyl) sulphide ((TMS)_2_S) was added drop-wise (at a rate of 7.93 mL h^–1^). After this addition was complete, the solution was left to stand for 2 min, and then 1.2 g of anhydrous zinc acetate was added. The solution was kept at 200 °C for 1 h and then cooled to room temperature. The particles were isolated by adding 40 mL of anhydrous degassed methanol and centrifuging. The supernatant was discarded, and 30 mL of anhydrous degassed hexane added to the remaining solid. The solution was allowed to settle for 5 h and then centrifuged again. The supernatant liquid was collected and the remaining solid was discarded. The quantum efficiencies of the final isolated indium-based nanoparticle material was approximately 85% in cyclohexane.

### Preparation of antibacterial polymers

Green emitting cadmium-free indium-based QDs (CFQD® nanoparticles) and crystal violet dye (CV) were embedded into polyurethane, a polymer widely used for medical device applications *via* the ‘swell-encapsulation-shrink’ dipping technique.^78^ Stock solutions of QDs in cyclohexane (2 mg mL^–1^) and CV in dichloromethane (1 mM) were used to prepare dipping solutions of the following final concentrations: 1 mg mL^–1^ QD + 0.5 mM CV, 1 mg mL^–1^ QD, and 0.5 mM CV in a solvent system of 1 : 1 cyclohexane : dichloromethane (Cy/DCM). Polymer squares (1 cm^2^) were dropped into each solution and left to swell in the dark for 24 h inside a closed bottle containing 10 mL of dipping solution. Subsequently, samples were left to dry in the dark at room temperature for 24 h, washed with dH_2_O and air dried. To produce polymer samples with QDs and CV (QD + CV PU), QDs only (QD PU) and CV only (CV PU) respectively. As controls, polymer samples were swollen in neat 1 : 1 Cy/DCM solvent.

### Material characterisation

Prior to transmission electron microscopy (TEM) imaging of quantum dots, CFQD® nanoparticles suspended in cyclohexane were drop cast onto 300 mesh carbon coated copper grid (Agar Scientific) and air-dried. High resolution (HR)-TEM images were acquired using a JEOL 2100 transmission electron microscope (TEM) with a LaB6 source operating at an acceleration voltage of 200 kV. Micrographs were taken on a Gatan Orius charge-coupled device (CCD) camera with Digital Micrograph software. Particle size analysis was carried out using Gatan Suite software. UV–vis absorption spectra of suspensions and polymer substrates were recorded using the Shimadzu UV-2600 Spectrophotometer (300–800 nm range). Emission spectra of suspensions was recorded with the Horiba FMax4 Fluorimeter (405–790 nm) at the excitation wavelength of 400 nm or 532 nm. For polyurethane substrates, a longpass Schott filter was installed into the fluorimeter to reduce scattered light from the excitation wavelength and samples were mounted diagonally at 45° prior to emission spectra measurement.

### Time-resolved lifetime measurements

Photoluminescence lifetime of QDs was measured using time-correlated single photon counting (TCSPC). Solutions of QDs and QD + CV complexes at various CV concentrations (fixed QD concentration) were prepared and placed in a 1 cm optical path quartz cuvette. A pulsed laser diode module Edinburgh instrument Ltd, UK model EPL-405 was used to excite the samples at 405 nm at a 1 MHz repetition rate (EPL-405, Edinburgh Instruments Ltd, UK). The emission was detected using a fast multi-alkali photomultiplier module (model H5773–04, Hamamatusu Photonics K.K., Japan) *via* a long-pass filter (OG510, Schott, UK) and a monochromator (model M300, Bentham Instrument Ltd, UK). A Lyot depolarizer (Thorlabs Ltd, Ely, UK) was incorporated to minimise any polarisation anisotropy artefacts. TCSPC was carried out using a PC-mounted TCSPC board (TimeHarp 260, PicoQuant GmbH, Germany) and lifetimes were derived using Fluofit software (PicoQuant GmbH, Germany). The Instrument Response Function (IRF) was obtained from a non-fluorescent scattering Ludox solution (Sigma-Aldrich, Gillingham, UK). Optimum fitting with minimisation of the residuals was confirmed using a Chi-squared value *χ*^2^ < 1.4. The amplitude-weighted mean lifetimes were derived using bi-exponential fitting. The photoluminescence lifetime of QDs was also measured after the incorporation of QDs and/or CV in the polymer. The substrates were mounted on microscope glass slides and signals were detected in the same manner as described for QD and QD + CV solutions above.

### Singlet oxygen phosphorescence

The singlet oxygen phosphorescence at 1270 nm of modified medical grade polyurethane incorporated with QDs and/or CV was detected using time-resolved photon counting. For detection in the near-IR, a thermoelectrically cooled photomultiplier (model H10330-45, Hamamatsu Photonics Ltd, Hertfordshire, UK) was used, and the emission was collected *via* a series of lenses from the cuvette in combination with a long-pass (950 nm cut-on, Andover Corp., USA) and a band-pass filter centred at 1270 nm (Interferenzoptik Electronik GmbH, Germany). Polyurethane samples were mounted diagonally in a quartz cuvette and irradiated using a 532 nm Nd:YAG laser (Lumanova GmbH, Germany) with the beam axis aligned at 45° to the surface plane of the sample in order to optimize detection of ^1^O_2_ within the polymer. The laser was pulsed at a repetition rate of 3 kHz and a pulse length of 3 ns, and a fast photodiode (1 ns rise time, Becker-Hickl, Germany) was used to synchronize the laser pulse with the photon counting detection system. Neutral density filters were used to attenuate the laser power to 2 mW. The photon counting equipment consisted of a PC-mounted multiscaler board (model MSA-300, Becker-Hickl, Germany) and a pre-amplifier (Becker-Hickl, Germany) which gave a resolution of 5 ns per channel. Time-resolved phosphorescence measurements were accumulated by the multiscaler board at a 0.1 μs bin width and the signals were analyzed using FluoFit software (PicoQuant GmbH, Germany) to extract the lifetime parameters.

### Bacterial strains

A MDR clinical strain of *Escherichia coli* (*E. coli* 1030) which produces both NDM-1 and OXA-48 carbapenemases, an environmental isolate of *Pseudomonas aeruginosa* (P12) recovered from a sink within the liver intensive care unit and a clinical isolate of *P. aeruginosa* (P1068) from a CF patient, were obtained from J. Wade, King's College Hospital, London. A clinical strain of epidemic methicillin-resistant *Staphylococcus aureus* (EMRSA 4742) used in the mechanistic study was obtained from P. Wilson, University College London Hospital.

Bacteria were stored at –70 °C in Brain–Heart Infusion broth (BHI, Oxoid) containing 20% (v/v) glycerol and propagated onto either MacConkey agar (MAC, Oxoid) in the case of *E. coli* and *P. aeruginosa* or mannitol salt agar (MSA, Oxoid) in the case of EMRSA, for a maximum of two subcultures at intervals of two weeks before reviving once more from freezer stocks.

### Antibacterial activity

Polymer substrates tested for antibacterial activity included:

• Quantum-dot and CV-encapsulated samples (QD + CV PU) – prepared by a 24 h immersion of polyurethane in a 1 : 1 hexane/DCM swelling solution containing a combination of red QDs and CV or green QDs.

• Quantum dot-encapsulated samples (QD PU) – Polyurethane immersed in a 1 : 1 cyclohexane/DCM swelling solution containing 1 mg mL^–1^ green-emitting QDs nanoparticles (QD PU) for 24 h.

• Crystal violet-encapsulated samples (CV PU) – Polyurethane squares were immersed in a 1 : 1 cyclohexane/DCM swelling solution containing 0.5 mM CV for 24 h.

• Control samples (control PU): polyurethane immersed in neat 1 : 1 cyclohexane/DCM solvent for 24 h.

The 1 cm^2^ squares of QD and CV encapsulated polyurethane (QD + CV PU), QD encapsulated polyurethane (QD PU), CV encapsulated polyurethane (CV PU) and solvent treated polyurethane (control PU) were tested against bacteria as follows:

To test each strain, BHI broth was inoculated with ∼3 bacterial colonies and cultured in air at 37 °C for 18 h with shaking, at 200 rpm. The bacterial pellet was recovered by centrifugation, (20 °C, 2867.2 g, 5 min), washed in PBS (10 mL), and centrifuged again to recover the pellet (20 °C, 2867.2 g, 5 min), and the bacteria were finally re-suspended in PBS (10 mL). The washed suspension was diluted 1000-fold to obtain an inoculum of ∼10^6^ CFU per mL (colony forming units per mL). In each experiment, the inoculum was confirmed by plating 10-fold serial dilutions on agar for viable counts. 25 μL of the inoculum was pipetted onto the surface of each polymer type and then the polymers were incubated at room temperature (18 h for *E. coli*, 24 h for *P. aeruginosa*), either in the light or in the dark. For light conditions, a white fluorescent tube light source (Osram 58 W/865 LUMILUX T8), giving an incident light intensity of ∼499 ± 19 lux, was employed.

After incubation, the inoculated samples were transferred to a tube containing PBS (450 μL) and mixed using a vortex mixer. The neat suspension and 10-fold serial dilutions were plated on agar for viable counts and incubated aerobically at 37 °C for 24 h (*E. coli* and *P. aeruginosa*) and 48 h (EMRSA). Each experiment contained a minimum of two technical replicates and the experiments were carried out at least three times. Error bars represent the standard deviation from the mean, and significance was evaluated using the unpaired *t*-test. Differences were considered significant for *P* < 0.05.

### Reactive oxygen species generated by materials

To investigate the nature of ROS generated by the QD + CV PU materials against bacteria, a H_2_O_2_ scavenger (catalase, 400 U mL^–1^), a hydroxyl radical scavenger (mannitol, 33 mM) and a ^1^O_2_ scavenger (l-histidine, 4 mM) were added to the bacterial suspension (methicillin-resistant *S. aureus*, EMRSA 4742) and exposed to the polyurethane substrates to deactivate the respective ROS emanating from the polymer surface. Catalase, mannitol and l-histidine were purchased from Sigma-Aldrich, UK and filter sterilised using a 0.2 μm PES syringe filter (VWR, UK). The control PU, CV PU and QD + CV PU substrates were tested against EMRSA 4742 illuminated with a white light source (6600 ± 900 lux) intensity for a period of 30 min, using the protocol described above. As a control, a separate experiment was conducted in the absence of any ROS quenchers.

## Conflicts of interest

There are no conflicts of interest to declare.
